# Identifying the primary outcome for a randomised controlled trial in rheumatoid arthritis: the role of a discrete choice experiment

**DOI:** 10.1186/s13047-017-0240-3

**Published:** 2017-12-15

**Authors:** Eugena Stamuli, David Torgerson, Matthew Northgraves, Sarah Ronaldson, Lindsey Cherry

**Affiliations:** 1 York Trials Unit, Department of Health Sciences, University of York, York, YO10 5DD UK; 2 Solent NHS Trust & University of Southampton, Faculty of Health Sciences, B45, Southampton, SO17 1BJ UK

**Keywords:** Rheumatoid arthritis, Discrete choice experiment, Choice of outcome measure, Randomized clinical trial

## Abstract

**Background:**

This study sought to establish the preferences of people with Rheumatoid Arthritis (RA) about the best outcome measure for a health and fitness intervention randomised controlled trial (RCT). The results of this study were used to inform the choice of the trial primary and secondary outcome measure.

**Methods:**

A discrete choice experiment (DCE) was used to assess people’s preferences regarding a number of outcomes (foot and ankle pain, fatigue, mobility, ability to perform daily activities, choice of footwear) as well as different schedules and frequency of delivery for the health and fitness intervention. The outcomes were chosen based on literature review, clinician recommendation and patients’ focus groups. The DCE was constructed in SAS software using the D-efficiency criteria. It compared hypothetical scenarios with varying levels of outcomes severity and intervention schedule. Preference weights were estimated using appropriate econometric models. The partial log-likelihood method was used to assess the attribute importance.

**Results:**

One hundred people with RA completed 18 choice sets. Overall, people selected foot and ankle pain as the most important outcome, with mobility being nearly as important. There was no evidence of differential preference between intervention schedules or frequency of delivery.

**Conclusions:**

Foot and ankle pain can be considered the patient choice for primary outcome of an RCT relating to a health and fitness intervention. This study demonstrated that, by using the DCE method, it is possible to incorporate patients’ preferences at the design stage of a RCT. This approach ensures patient involvement at early stages of health care design.

**Electronic supplementary material:**

The online version of this article (10.1186/s13047-017-0240-3) contains supplementary material, which is available to authorized users.

## Background

Rheumatoid arthritis (RA) is a systemic, disabling disease which has a prevalence of 0.44% for men and 1.16% for women in the UK [1], equating to around 400,000 adults having the condition in 2006 [2]. Approximately 90% of people with RA eventually develop symptoms related to the foot or ankle [3]. The occurrence of RA in the feet can reduce mobility, place restrictions on daily life and cause pain for patients, hence affecting quality of life [4]. In addition, those with impaired walking ability due to RA of the feet are also at increased risk of falling and hence, fall-related injuries such as fractures may occur. The feet are affected early in the disease course of RA, with pain or damage leading to altered gait [5, 6].

Currently there is no treatment offered which specifically aims to help maintain or improve walking ability for people with RA of the foot or ankle. Benefits have been shown for gait rehabilitation strategies (i.e. a range of techniques to improve walking) in people who have other conditions where their walking is affected [7, 8]. However, there have been no randomised controlledtrials (RCTs) conducted in this area to assess it fully. It would therefore be valuable to design and undertake a RCT in order to assess the effectiveness of a health and fitness intervention intended to facilitate gait rehabilitation in people with RA of the foot or ankle. The health and fitness intervention will adopt the nature of an existing programme, ESCAPE [9], which enables self-management and coping with arthritic pain using exercise, and is widely used for people with chronic joint pain.

A crucial element for the conduct of a rigorous study is the collection of the most appropriate outcome on as many trial participants as possible [10]. The decision as to which outcome(s) should constitute the primary outcome(s) of an RCT - and which are secondary outcomes- is made by the study investigators, with clinical advice where required (e.g. through Delphi panels of clinicians to reach consensus on the most appropriate outcome). This choice is often driven by what has been used in previous studies or through a literature review and what clinicians perceive as being most clinically relevant to patients, once the relationship between the intervention component and the outcome measure, together with the theories concerning mode-of-action of the intervention have been established. More recently, and in accordance with guidance from the patient group INVOLVE [11], the views of patients are being included in the choice of outcome measurements. This approach ensures the patient involvement in the health care decision making process, which is in line with governments initiatives in many health care systems [12]. For greater benefits, both in financial and clinical outcomes terms, the involvement of the patients is thought to be pertinent not only at the care level, but also at the research and design level [13]. This is clearly defined in the aims of the National Health System (NHS) in the UK [14] where the patients are invited to be involved in the co-design, co-commissioning and co-production of health care.

This approach is potentially very important as a patient perspective can not only identify key outcomes but can be used to weight or sequence key outcomes in order of their perceived importance. In this paper we demonstrate a method – the Discrete Choice Experiment (DCE) - of identifying and then prioritising the key outcomes that are important to patients, once clinically relevant outcomes have been identified and mechanisms of change are known. This enables the trialists to design research around the primary outcome supported by the patient group of interest and include relevant secondary outcome measures. The primary outcome measure for clinical trials is, arguably, not directly or solely decided by patient preferences. Some outcome measures, such as survival, might be straight forward to choose but often there are trade-offs between a treatments effect, its intensity and modality and patients are probably best placed to make a judgement as to the priority of these different aspects. The DCE can combine the use of qualitative methods (i.e. interviewing patients as to their choice of attributes) with a quantitative methodology to prioritise these attributes. As well as aiding the identification of key outcomes, a DCE can also help researchers refine their choice of intervention by asking potential participants to prioritise the various treatment options so that when there are mutually exclusive choices (e.g. group versus one-to-one therapy) the DCE can inform treatment construction.

A DCE is a quantitative method for eliciting preferences regarding alternative scenarios or options. Participants are presented with alternative hypothetical scenarios and are asked to indicate their most preferred option, with each option involving several attributes (outcomes) with different levels. DCEs have been commonly used in the field of health economics to address a wide range of policy questions [15]. By conducting a DCE at an early stage in the RCT design, it can help ensure that the primary outcome of the trial is relevant to the patient, in this instance those with a diagnosis of RA. The aim of this study was to use a DCE in order to assess the relative importance of different outcomes, as well as the nature and schedule of the intervention, with the findings helping to guide the design of an RCT in this area.

## Methods

### Outcomes identification

The Leeds Foot Impact Scale (LFIS) [16] served as the initial tool for the identification of the outcomes related to foot and ankle RA. LFIS is a quantitative measure of foot impacts associated with RA. It includes 2 subscales, and 51-items covering the domains of impairments/shoes and activities/participation. LFIS is considered a reliable, disease-specific scale to measure the outcome of interventions for studies in the foot and ankle RA field [16]. A literature review was then conducted with the aim to supplement and support the choice of the attributes from the LFIS. It sought to find the most frequently reported outcomes in RA of foot and ankle patients. The review was conducted using Pubmed and Embase databases, using broad search terms like “rheumatoid arthritis” and “patient reported outcomes”.

The identified outcomes were used to populate the list of attributes to be included in the DCE. Five attributes were selected that were deemed to be the most important outcomes from patients’ and clinical perspectives, and reported in the literature.

(i) *Pain.* Changes in pain have been identified as the most important reported outcome from a patient’s perspective both clinically and in RA trials [17–20]. Furthermore, the inclusion of pain as one of the seven core set of outcomes of disease activity as identified by both the American College of Rheumatology (ACR) [21] and European League against Rheumatism (EULAR) [22] emphasises its importance from a clinician’s perspective. Using a nominal group technique (*n* = 26), Sanderson et al. [17] refined a previously established list of 63 important patient reported outcomes [23] and identified reducing pain as the top priority outcome ahead of limiting joint damage, reducing fatigue, improving ability to perform daily living activities and mobility. Similarly, studies by Gossec et al. [18], Heiberg et al. [19] and ten Klooster et al. [20] all identified changes in pain as the predominant patient reported outcome.
(ii) *Fatigue.* Fatigue has been reported to be an under reported attribute in RA [24] however in 2007, as a result of a patient focus group discussions, it was recommended by Outcome Measures in Rheumatology (OMERACT [25]) that measurement of fatigue was included alongside the 7 items identified in the ACR core set [26]. This recommendation was subsequently supported by both ACR and EULAR in 2008 [27]. A number of qualitative studies have identified fatigue as one of the most important patient perceived outcomes [17, 18, 26, 28]. Both Sanderson et al. [17] and Gossec et al. [18] reported reduced fatigue as the third most important patient perceived outcome behind pain and either joint damage [17] or physical disability [18].
(iii) *Mobility.* Given the development of foot and ankle RA is associated with decreased walking speed, increased periods of double stance and reduced joint range of motion [29], changes in mobility represents an important patient reported outcome in RA patients. Focus group interviews with Swedish RA patients (*n* = 25) identified increased mobility along with reduced pain, stiffness and fatigue as important contributors to improved physical capacity [28]. In two studies [19, 20] using the AIMS2 questionnaire (which focuses on 12 areas of health – mobility, walking and bending, hand and finger function, arm function, self-care, household tasks, social activity, support from family, arthritis pain, work, level of tension and mood) walking and bending, and mobility were identified as being in the top five priority areas for health improvement. Heiberg & Kvien [19], which surveyed 1024 Norwegian RA patients, identified walking and bending as the third preferential area (33.3%) for health improvement, with mobility ranked fifth (23.9%). Similarly, ten Klooster et al. [20] reported over a 1 year period of active RA treatment (*n* = 173); priorities for improvement did not vary greatly with walking and bending (42.2%), and mobility (32.9%) ranked as the third and fourth preferential areas of improved health.
(iv) *Ability (or lack of) to perform activities of daily living*. Along with the mobility issues discussed previously, the ability (or lack of) to perform activities of daily living (e.g. work and household tasks, family and leisure activities) is another important area highlighted by RA as impacting on patients lives [17, 19, 28]. Qualitative studies by Sanderson et al. [17] and Ahlmen et al. [28] both highlighted a decline in the ability to perform everyday activities impacted on the independence of RA patients. Furthermore, a decline in both ability to perform activities of daily living and mobility were identified as components that contribute to increased functional disability [17].
(v) *Choice of footwear.* Although choice of footwear has not previously been identified as a priority outcome in RA patients, the majority of studies have focused on RA as an overarching disease affecting the whole body rather than focusing specific regions (e.g. ankle/ft, hands). Rheumatoid arthritis of the ankle and foot can result in specific limitations with the development of joint stiffness and deformity acting as factors that can limit the choice of suitable footwear available to the patient [16]. Whilst correctly fitted therapeutic footwear and orthotics are thought to reduce pain, improve mobility and preserve foot function [30, 31], ill-fitting or incorrect footwear can exacerbate the symptoms [32]. Furthermore, despite the potential therapeutic benefits of specialist footwear, a reluctance to wear them can exist, especially in female patients, where the appearance of the footwear is reported to potentially have a negative effect on body image [33]. Restriction to footwear choice therefore represents an important factor in foot and ankle RA patients.

One additional attribute was a “process” attribute and was chosen to elicit patients’ preference for the schedule of the intervention. Similar to choosing an outcome as primary outcome of the RCT, the attribute related to the schedule of the intervention would feed into the design of the RCT. There were two components for this attribute: whether the intervention was delivered on a one-to-one or on a group basis, and the frequency of attendance i.e. whether it was delivered once a week for 12 weeks or twice a week for 6 weeks.

All the attributes, with the exception of the schedule of the intervention, were described as three level attributes ranging from extremely bad to extremely good states (Table 1).

**Table 1:** List of attributes and levels

Attributes	Levels	Variable name used in the model
Pain	My feet are not painful when I walk My feet are somewhat painful when I walk My feet are extremely painful when I walk	pain_2 pain_1 Reference
Mobility (e.g. walking, climbing stairs)	I have no problems with mobility I have some problems with mobility I have extreme problems with mobility	mobility_2 mobility_1 Reference
Doing everyday things / activities of daily living (e.g. work, study, housework, family or leisure activities)	I have no problems with doing everyday activities I have some problems with doing everyday activities I have extreme problems with doing everyday activities	daily_activities_2 daily_activities_1 Reference
Fatigue	I usually do not feel tired after walking I usually feel moderately tired after walking I usually feel extremely tired after walking	fatigue_2 fatigue_1 Reference
Shoes (choice of footwear)	My walking ability is not affected by the footwear I choose. My walking ability is moderately affected by the footwear I choose. My walking ability is greatly affected by the footwear I choose	shoes_2 shoes_1 Reference
Health and fitness – exercise supervised by a physiotherapist or podiatrist	To improve my walking, I will go for one-to-one supervised exercise, twice a week for 6 weeks (1) To improve my walking, I will go for one-to-one supervised exercise, once a week for 12 weeks (2) To improve my walking, I will go for supervised exercise as part of a group twice a week for 6 weeks. (3) To improve my walking, I will go for supervised exercise as part of a group once a week for 12 weeks. (4)	health_fitness_3 health_fitness_2 health_fitness_1 Reference

### Qualitative work

In addition to the literature review, consultation with clinicians and stakeholder groups, through focus groups and interviews, contributed to the validation and the refinement of the list of attributes and the respective levels. Three clinicians, working across primary and secondary care services within Wessex healthcare region, were involved. The attributes were also discussed with patients from two NHS services who were invited by way of email to an established patient advice group to contribute to this work. Two key lay members were subsequently identified and had further specific input into this work. All groups involved were asked to generally comment on whether attributes chosen represent the most important outcomes for patients with foot and ankle RA. The list was finalized through an iterative process of adding/removing attributes and improving wording of the attributes and levels, and seeking consensus amongst clinicians, patients and lay members.

### Design of the experiment

The number of the attributes chosen for this DCE and the respective levels would result in 3^5*4 = 972 profiles or scenarios (i.e. all possible combinations of five three-level and one four-level attributes) and 471,906 choice pairs [i.e. (972 × 971)/2]. A D-efficient (or D-optimal) design was chosen to produce a manageable number of choice pairs, yet satisfying statistical efficiency. D-Optimality characterizes the selection of a special set of experiments which fulfils a given criterion. In a D-optimal design the determinant of the covariance matrix is minimized. Hence, there is minimum variation around the parameter estimates due to minimized estimated standard errors [34, 35].

The experimental design was created in SAS software (version 9.4) with the use of in-built macros [36]. % ***mktruns*** macro creates the candidate alternatives and recommends possible design sizes. No prior assumptions were made about the parameters to be estimated; hence they were set to be zero. A number of designs with varying number of choice sets and respective D-efficiency criterion were explored with the aim of choosing the design which provided the best compromise between statistical efficiency and minimizing the cognitive burden to the respondents, due to the length of the questionnaire. A 18-choice set design was chosen. One choice set was repeated to test the consistency of the responses. Only main effects are estimated by this experimental design; inclusion of interaction terms would have resulted in a larger number of choice sets and lengthier questionnaire.

### The choice task and data collection

A market research company, Research Now, which maintains large panels of respondents, was employed for the data collection. The company recruited the respondents, designed the web questionnaire based on the experimental design, and constructed the survey website. One hundred RA patients aged 18 or over and based in the UK were recruited to complete the survey. Symbolic remuneration fees were given to the respondents. In addition to completing the DCE, participants provided information on their age, occupation and region of residence in the UK.

An information page and instruction on how to complete the survey were provided in the opening page of the web survey. Participants were presented with the list of choice sets (in random order) and were asked to choose the most preferred scenario between the two in each choice set, not the option they felt closely reflected their current situation. Participants were familiarized with the task by responding to two “warm-up” choice sets.

The participant information sheet and an example DCE choice set are provided in Fig. S1 and Fig. S2 of the Additional file 1 respectively.

### Data analysis

The analysis of the DCE data is based on the random utility framework, where the respondent is assumed to choose within a choice set the alternative that will maximise their utility. The utility function is specified as:

1 $$ {\mathrm{U}}_{\mathrm{iq}}=\mathrm{V}\ \left({\mathrm{X}}_{\mathrm{iq}},\upbeta \right)+{\upvarepsilon}_{\mathrm{iq}} $$

[15] where U_iq_ is the utility of the ith alternative for the qth individual. The utility is comprised of a systematic component, V (Xiq, β) specified as a function of the attributes of the alternatives, and a random component ε_iq_ which captures the unmeasured variation in preferences.

Three econometric models were used for the analysis of the DCE data based on different assumptions: a conditional logit model (CLOGIT), which is considered the “workhorse” for the analysis of DCE data [37], the mixed logit (MXL) and the generalized multinomial logit (GMNL) model. The MXL model accounts for unobserved *preference (taste) heterogeneity* [38] and GMNL model accounts for both *preference and scale heterogeneity* [39], while relaxing the assumptions of the conditional logit model. A number of different models were fitted in the GMNL framework. Additional notes are provided in the Additional file 2.

Akaike information criterion (AIC) was used to assess the statistical fit of the models. Lower AIC values indicate better fit. Combining the criteria of 1) better statistical fit with 2) the possibility of accounting for different types of heterogeneity (taste and/or scale heterogeneity), would lead to using the results of the most appropriate model.

Simple descriptive statistics were generated for the demographic variables.

All the analyses were conducted in STATA statistical package (version 14SE). The clogit procedure was used for the conditional logit model, the mixlogit command [40] was used for the MXL model and the GMNL model was operationalized with the use of the gmnl command [41].

### Relative impact of attributes

In order to examine which attribute/outcome was the most significant for the respondents the *partial log-likelihood method* was used [42]. The analysis was conducted by systematically re-estimating the models after dropping from the estimation one attribute at a time, and noting the respective log-likelihood value of the model. The partial effect on the log-likelihood i.e. the difference between the log-likelihood of the model with all the predictors and that of the model with one omitted variable, was used to order the attributes by their impact. In other words, the attribute with the largest partial effect-change in log-likelihood, is considered the most important one compared to the other attributes included in the DCE. The result of this analysis would feed directly into the decision for choosing the primary outcome for the trial design.

## Results

### Sample characteristics

The sample characteristics are presented in.

Table 2. A total of 100 respondents were recruited across the UK. There was an equal gender representation in the sample. The mean age of the respondents was 57 years and the majority (41%) belonged to the E category of the social grade classification i.e. state pensioners, casual and lowest grade workers or unemployed with state benefits.

**Table 2:** Demographic characteristics

Total sample	*N* = 100
Gender (%females)	51
Mean (SD) age in years	57 (12.3)
Age groups (%)	
18–24	0
25–34	3
35–44	14
45–54	20
55–64	34
65+	29
Social grade (%)	
A - Higher managerial, administrative and professional	7
B - Intermediate managerial, administrative and professional	18
C1 - Supervisory, clerical and junior managerial, administrative and professional	11
C2 - Skilled manual workers	15
D - Semi-skilled and unskilled manual workers	8
E - State pensioners, casual and lowest grade workers, unemployed with state benefits only	41

### Models results

Table 3 presents the results from the CLOGIT and MXL models. None of the models fitted in the GMNL framework were significant, based on the Wald test, hence the results are not included here. Additional file 3 provides more details on the results of different models. The goodness-of-fit measures (Table 4) demonstrated little difference between the CLOGIT and MXL model, with slight advantage for MXL. This probably is indicative of the fact that the preference heterogeneity that would be captured by the MXL does not impact hugely on the overall results.

**Table 3:** Results of conditional logit and mixed logit analysis

Variable (name used in the model)	Conditional logit model	Mixed logit model
	Coefficient (SE)	Coefficient (SE)
My feet are somewhat painful when I walk (pain_1)	0.389 (0.069)***	0.474 (0.082)***
My feet are not painful when I walk (pain_2)	0.44 (0.069)***	0.536 (0.12)***
I have some problems with mobility (mobility_1)	0.350 (0.069)***	0.443 (0.079)***
I have no problems with mobility (mobility_2)	0.176 (0.069)*	0.139 (0.117)
I have some problems with doing everyday activities (daily_activities_1)	0.195 (0.069)**	0.264 (0.08)***
I have no problems with doing everyday activities (daily_activities_2)	0.27 (0.068)***	0.396 (0.098)***
I usually feel moderately tired after walking (fatigue_1)	0.167 (0.069)*	0.235 (0.079)**
I usually do not feel tired after walking (fatigue_2)	0.045 (0.068)	0.052 (0.081)
My walking ability is moderately affected by the footwear I choose (shoes_1)	0.161 (0.069)*	0.223 (0.079)**
My walking ability is not affected by the footwear I choose (shoes_2)	0.104 (0.069)	0.129 (0.092)
To improve my walking, I will go for supervised exercise as part of a group twice a week for 6 weeks (health_fitness_1)	−0.027 (0.085)	−0.057 (0.102)
To improve my walking, I will go for one-to-one supervised exercise, once a week for 12 weeks (health_fitness_2)	0.124 (0.084)	0.149 (0.099)
To improve my walking, I will go for one-to-one supervised exercise, twice a week for 6 weeks (health_fitness_3)	0.131 (0.084)	0.173 (0.097)
*Standard deviations*		
pain_1		−0.231 (0.147)
pain_2		0.989 (0.121)***
mobility_1		0.003 (0.196)
mobility_2		0.924 (0.119)***
daily_activities_1		0.123 (0.136)
daily_activities_2		0.626 (0.11)***
fatigue_1		0.004 (0.138)
fatigue_2		−0.198 (0.141)
shoes_1		0.043 (0.098)
shoes_2		−0.481 (0.098)***
health_fitness_1		0.314 (0.158)*
health_fitness_2		−0.23 (0.194)
health_fitness_3		−0.010 (0.174)
Number of observations	3600	3600
chi-squared	108.661	119.858
Model degrees of freedom	13	13

* *p* < 0.05, ** *p* < 0.01, *** *p* < 0.001

**Table 4:** Goodness-of-fit of the two models

Measure	Conditional logit model	Mixed logit model
Log likelihood	−1193.335	−1133.406
AIC	2412.669	2318.811
BIC	2493.122	2479.717

The coefficients in Table 3 indicate the relative importance, or the utility increase, of moving from the reference state to the particular level of the attribute. In both models, significant coefficients and their positive signs denote a preference of the respondents for the particular state versus the reference state, which is always the extremely bad state. Hence, respondents prefer good states rather than bad states, which is natural and intuitive. When the coefficients are non-significant (as denoted by the *p*-values), there is no evidence of explicit preference of the respondents for one state/schedule versus the reference state. This is strongly highlighted in the case of the health and fitness intervention for gait rehabilitation where the respondents are indifferent towards different combinations of the frequency of the intervention (once a week for 12 weeks or twice a week for 6 weeks) and the nature of the intervention (one-to-one or group intervention). This is observed in both models results.

For the attributes pain and daily activities, respondents attach greater importance to achieving the best health states compared to moderately impaired states. This is denoted by the fact that the coefficients are larger for the perfect health state vs the reference health state, compared to the moderately impaired health states vs the reference health state. This is not the case, however, for the attributes mobility, fatigue and choice of shoes. For these attributes, the magnitude of the coefficients indicate that respondents attach greater importance to moving from the extremely impaired state to the mildly impaired state than to moving from the extremely impaired state to the perfect state. For example, in the conditional logit model results, the coefficient (i.e. the utility increase) for “I have no problems with mobility” is smaller than that for “I have some problems with mobility” (0.176 vs 0.350).

The results from both models are broadly comparable with respect to the signs and significance of the coefficients (Table 3). The only difference is for the preference on the state describing no problems with mobility versus the reference state which is having extreme problems with mobility. This is not significant in the mixed logit model. For the mixed logit model, the significance of the standard deviation around the mean values of the coefficients signifies the existence of unobserved preference heterogeneity in the data. Preference heterogeneity exists in the choice for most of the “perfect” health states versus the extremely bad states (e.g. “My feet are not painful when I walk” compared to “My feet are extremely painful when I walk”). This is not the case for the moderate states versus the extremely bad states. The non-significance of the standard deviations may be an indication of low heterogeneity of preferences among respondents for the specific attribute levels.

The results of the analysis on the relative impact of the attributes are presented.

Table 5. The outcomes are listed by decreasing order of impact on the overall log likelihood of the model. This can be interpreted as the attributes higher up in the list being the most important. The results demonstrated that moving from extreme pain to no pain was the most significant outcome for both models; it accounts for 27% of the log-likelihood, or 49% for both levels in the CLOGIT model.

**Table 5:** Hierarchical importance of attributes based on partial log-likelihood analysis

Conditional logit model				Mixed logit model			
Attribute level excluded from the analysis (by order of impact)	Log likelihood	Partial effect- change in log likelihood	Relative effect - %sum of change in log likelihood	Attribute level excluded from the analysis (by order of impact)	Log likelihood	Partial effect-change in log likelihood	Relative effect -%sum of change in log-likelihood
None	−1193.335			None	−1133.406		
Pain_2	−1213.810	−20.475	0.276	Pain_2	−1190.501	−57.095	0.264
Pain_1	−1209.571	−16.236	0.219	Mobility_2	−1167.962	−34.556	0.160
Mobility_1	−1206.337	−13.002	0.175	DActivities_2	−1157.175	−23.769	0.110
DActivities_2	−1201.174	−7.839	0.106	Pain_1	−1156.766	−23.360	0.108
DActivities_1	−1197.358	−4.023	0.054	Mobility_1	−1155.712	−22.306	0.103
Fatigue_1	−1196.652	−3.317	0.045	DActivities_1	−1146.207	−12.801	0.059
Mobility_2	−1196.297	−2.962	0.040	Fatigue_1	−1144.621	−11.215	0.052
Shoes_1	−1196.081	−2.746	0.037	Shoes_1	−1144.179	−10.773	0.050
HF_3	−1194.546	−1.211	0.016	Shoes_2	−1142.456	−9.050	0.042
HF_2	−1194.473	−1.138	0.015	HF_1	−1138.126	−4.720	0.022
Shoes_2	−1194.413	−1.078	0.015	Fatigue_2	−1137.004	−3.598	0.017
Fatigue_2	−1193.553	−0.218	0.003	HF_2	−1135.117	−1.711	0.008
HF_1	−1193.386	−0.051	0.001	HF_3	−1134.987	−1.581	0.007

*Notes*:

_1 refer to “moderate” states

_2 refer to “perfect” or “no problems” states

Coefficients calculated based on the “extremely bad” state being the baseline

HF_1: ‘To improve my walking, I will go for supervised exercise as part of a group twice a week for 6 weeks’

HF_2: ‘To improve my walking, I will go for one-to-one supervised exercise, once a week for 12 weeks’

HF_3: ‘To improve my walking, I will go for one-to-one supervised exercise, twice a week for 6 weeks’;

Baseline is: ‘To improve my walking, I will go for supervised exercise as part of a group once a week for 12 weeks’

## Discussion and conclusions

This study is one of the first to demonstrate the use of DCE for establishing preferences of people with RA for different outcomes and schedule of a health care intervention intended for gait rehabilitation. The results from this DCE were used as a guide for the design of a RCT, where the most highly valued outcome from this exercise would inform the choice of the primary outcome in the trial. In this instance, patients weighted foot and ankle pain as the most important outcome, with mobility being nearly as important, and measures of these should be either the primary outcome of the trial or a key secondary outcome. This approach is ensuring the patient involvement at early stages of health care design, evaluation and decision making.

One hundred people with RA completed the DCE and the results indicate that people value mostly the reduction in pain. This result was consistent across different econometric models used for analysing the DCE data, which is reassuring that the findings of this analysis are robust and reliable. Different econometric models were used to relax restrictive assumptions on the properties of the discrete choice data, and take into account scale or preference heterogeneity. From the analysis, there did not appear to be sufficient evidence of scale heterogeneity or large preference heterogeneity. If large preference heterogeneity exists, then this has implications for the policy decisions. For example, the treatment schedule could be more flexible, to cater for the range of preferences expressed.

Nevertheless, the results demonstrated that the people with RA have no explicit preference for the different health and fitness intervention schedules: there was no preference between attending the exercises on a one-to-one or group basis, and similarly on the frequency of the sessions. For the design of the RCT, this is an important aspect as other elements such as the cost of each schedule or the availability of the practitioners in the different sites might drive the choice of the service design. In terms of the outcomes, the respondents preferred good states rather than bad states, which is an expected and intuitive response.

For a set of three attributes, mobility, fatigue and choice of shoes, participants appear to gain larger utility from mildly impaired states (vs. reference state) than from perfect states (vs. reference state). This is denoted by the magnitude of the coefficients in both models. The findings in this case appear counter-intuitive as one would expect the opposite. However, a number of reasons might lie behind these results. Firstly, the adaptation effect, where people are used to living in an impaired state, might play a role in the respondents’ decision making process. Secondly, a pragmatic approach of the respondents that live daily with RA is probably evident here in the sense that they are aware of and complacent with the idea that no intervention will ever make their health state a perfect one, due to the nature of the disease itself. Thirdly, there might have been an overlap in the interpretation of certain attributes. For example, for as long one can perform their daily activities, the level of mobility is not of concern to them. Again, the adaptation effect, where people can perform their daily activities despite low levels of mobility. Additionally, the counter-intuitive results might be indeed a consequence of other study limitations.

One of the study limitations relates to the fact that the experimental design and the model fitting assumed only main effects; possible interaction effects were not taken into account. This might not fully reflect the clinical reality as for example, one could argue than the level of pain and the ability to do daily activities would have an inverse relationship. However, the decision to include only main effects in the DCE design was made to avoid an overly long and burdensome questionnaire for the respondent which would have resulted from the inclusion of the interaction effects.

Responses for this study were collected by using an internet panel. Under ideal conditions, with no time or budget constraint, the survey would have taken place on a face-to-face basis between the researcher and the respondents. By using an internet panel, there is an element of the respondents not fully understanding the task or rushing through the completion of it. The time respondents took to answer the questions, and the impact this had on the results is part of a separate study/publication. The sample size could potentially be considered another limitation of the study. Although having a sample size of 100 is considered satisfactory for this type of survey, a larger sample size might have revealed scale heterogeneity or larger preference heterogeneity.

A number of studies have assessed patients’ preferences in RA, ranging from variability in treatment preferences between racial groups [43], preferences for treatments with varying risk profiles by treatment [44] and preference for RA treatments based on the route of administration, the benefits and side effects [45]. The approach in this study is different and, to our knowledge, has not been applied before. This study has demonstrated that it is possible to include patients’ preferences at the RCT design stage to enable better definition of the treatment plan and to identify the primary outcome. We recommend that where there is uncertainty in either or both the treatment pathways and outcomes a DCE is undertaken before the RCT design is completed.

## Additional files

Additional file 1: Figure S1. Participant information sheet. **Figure S2.** Example DCE choice set. (DOCX 17 kb)

Additional file 2: Additional Notes on Data analysis. (DOCX 21 kb)

Additional file 3: Additional Notes on Results. (DOCX 21 kb)

## Abbreviations

ACR: American College of Rheumatology
AIC: Akaike information criterion
CLOGIT: Conditional logit model
DCE: Discrete choice experiment
EULAR: European League against Rheumatism
GMNL: Generalized multinomial logit
MXL: Mixed logit
NHS: National Health System
OMERACT: Outcome Measures in Rheumatology
RA: Rheumatoid arthritis
RCT: Randomised controlled trial
