# Supplementary material for: Identifying the primary outcome for a randomised controlled trial in rheumatoid arthritis: the role of a discrete choice experiment
Source: J Foot Ankle Res. 2017 Dec 15;10:57. doi: 10.1186/s13047-017-0240-3 (PMC5732456; doi:10.1186/s13047-017-0240-3)
Supplement: Supplementary file 1 — Participant information sheet. Figure S2. Example DCE choice set. (DOCX 17 kb) [file 13047_2017_240_MOESM1_ESM.docx]

**Journal of foot and ankle research**

**Additional file 1**

**Identifying the primary outcome for a randomised trial in rheumatoid arthritis: the role of a discrete choice experiment.**

**Eugena Stamuli^1^, David Torgerson^1^, Matthew Northgraves^1^, Sarah Ronaldson^1^, Lindsey Cherry^2^**

1. York Trials Unit, Department of Health Sciences, University of York, York YO10 5DD, UK

2. Solent NHS Trust & University of Southampton, Faculty of Health Sciences, B45, Southampton, SO17 1BJ, UK

**Corresponding author**

Eugena Stamuli

[eugena.stamuli@york.ac.uk](mailto:eugena.stamuli@york.ac.uk)

Tel: +44(0)1904 321871

Figure S1: Participant information sheet

| **What outcomes are important when assessing gait rehabilitation interventions for patients with rheumatoid arthritis of the foot or ankle?**  We are investigating the impact of a gait rehabilitation programme (range of techniques to improve walking) for people with rheumatoid arthritis of the foot or ankle.  In particular we are looking at the effect on people's walking ability and health-related quality of life. We are interested in your views on which outcomes are most important to you based on your experience of rheumatoid arthritis. We are also interested in your preferences for the nature and the schedule that the rehabilitation programme should have.  You will be presented with eighteen choice sets which include pairs of options (option A and option B). Each option has six characteristics associated with it:  *Pain: which describes the level of pain one feels when walking*  *Mobility: which describes the ability to walk or climb stairs*  *Everyday activities: which describes the ability to do every day things such as work, study, housework, family or leisure activities.*  *Fatigue: which describes the level of tiredness one experiences when walking*  *Shoes: describes whether the ability to walk is affected by the choice of shoes*  *Health and fitness: describes the nature and the schedule of the rehabilitation programme.*  We would like you to choose which option you prefer out of a choice of two: option A or option B. Place a tick in one box from each set to indicate which you prefer.  Important: Please choose the option you would prefer, NOT the option you feel best describes your current situation.  Please view each choice set independently, there is no need to remember previous choices. There are no wrong or right answers |
| --- |

Figure S2: Example DCE choice set

|  | Option A | Option B |
| --- | --- | --- |
| **Pain** | My feet are extremely painful when I walk | My feet are somewhat painful when I walk |
| **Mobility (e.g. walking, climbing stairs)** | I have no problems with mobility | I have some problems with mobility |
| **Everyday activities (e.g. work, study, housework, family or leisure activities)** | I have some problems with doing everyday activities | I have extreme problems with doing everyday activities |
| **Fatigue** | I usually feel extremely tired after walking | I usually feel moderately tired after walking |
| **Shoes** | My walking ability is moderately affected by the footwear I choose | My walking ability is not affected by the footwear I choose |
| **Health and fitness** | To improve my walking, I will go for supervised exercise as part of a group once a week for twelve weeks | To improve my walking, I will go for one-to-one supervised exercise, twice a week for six weeks |
| **Please choose your preferred option (tick A or B)** |  |  |
