# Supplementary material for: Identifying the primary outcome for a randomised controlled trial in rheumatoid arthritis: the role of a discrete choice experiment
Source: J Foot Ankle Res. 2017 Dec 15;10:57. doi: 10.1186/s13047-017-0240-3 (PMC5732456; doi:10.1186/s13047-017-0240-3)
Supplement: Supplementary file 2 — Additional Notes on Data analysis. (DOCX 21 kb) [file 13047_2017_240_MOESM2_ESM.docx]

**Journal of foot and ankle research**

**Additional file 2**

**Identifying the primary outcome for a randomised trial in rheumatoid arthritis: the role of a discrete choice experiment.**

**Eugena Stamuli^1^, David Torgerson^1^, Matthew Northgraves^1^, Sarah Ronaldson^1^, Lindsey Cherry^2^**

1. York Trials Unit, Department of Health Sciences, University of York, York YO10 5DD, UK

2. Solent NHS Trust & University of Southampton, Faculty of Health Sciences, B45, Southampton, SO17 1BJ, UK

**Corresponding author**

Eugena Stamuli

[eugena.stamuli@york.ac.uk](mailto:eugena.stamuli@york.ac.uk)

Tel: +44(0)1904 321871

**Additional Notes on Data analysis**

Three econometric models were used for the analysis of the DCE data based on different assumptions.

1. Conditional logit model

This model is based on a number of restrictive assumptions i.e. the independence of irrelevant alternatives (IIA), the independent and identically distributed (iid) across observations error term and the lack of unobserved preference heterogeneity across respondents. These assumptions might not be appropriate in all the cases [[1](#_ENREF_1)], hence it can lead to biased results [[2](#_ENREF_2)] and subsequent policy implications from the application of the DCE conclusions. Two other models were used additionally to analyse the data

1. The mixed logit model (MXL) which accounts for unobserved *preference (taste) heterogeneity* [[3](#_ENREF_3)], and
2. The generalized multinomial logit (GMNL) which accounts for both *preference and scale* heterogeneity [[4](#_ENREF_4)], while relaxing the other two assumptions of the conditional logit model.

The scale heterogeneity is the result of the idiosyncratic error term being greater for some consumers/respondents than it is for others; in other words, the choice behaviour is more random for some respondents than it is for others [[5](#_ENREF_5), [6](#_ENREF_6)].

For the mixed logit model, all the independent variables were assumed to have random coefficients, which were specified to be normally distributed. The random coefficients were also set to be independent in the first instance and then correlated; hence two separate models were run: the first time the coefficients uncorrelated, then correlated.

A number of different models were fitted in the GMNL framework. Three different *scale heterogeneity multinomial logit models (S-MNL)* were fitted to test whether there exists evidence of scale heterogeneity in the data: 1) a model without alternative specific constant (ASC) 2) a model with fixed ASC and 3) a model with random ASC – in this case ASC captures preference heterogeneity [[5](#_ENREF_5)]. One additional analysis was conducted with the use of the GMNL specification (with uncorrelated coefficients), which accounts simultaneously for *scale and preference heterogeneity.*

References:

1. Train KE: *Discrete choice methods with simulation.* Cambridge university press; 2009.

2. Flynn TN, Louviere JJ, Peters TJ, Coast J: **Using discrete choice experiments to understand preferences for quality of life. Variance-scale heterogeneity matters.** *Soc Sci Med* 2010, **70:**1957-1965.

3. McFadden D, Train K: **Mixed MNL Models for Discrete Response.** *Journal of Applied Econometrics* 2000, **15:**447-470.

4. Fiebig DG, Keane MP, Louviere J, Wasi N: **The generalized multinomial logit model: accounting for scale and coefficient heterogeneity.** *Marketing Science* 2010, **29:**393-421.

5. Gu Y, Hole A, Knox S: **Fitting the generalized multinomial logit model in Stata.** *Stata J* 2013, **13:**382-397.

6. Louviere J, Street D, Carson R, Ainslie A, Deshazo JR, Cameron T, Hensher D, Kohn R, Marley T: **Dissecting the Random Component of Utility.** *Marketing Letters* 2002, **13:**177-193.
