# Supplementary material for: Identifying the primary outcome for a randomised controlled trial in rheumatoid arthritis: the role of a discrete choice experiment
Source: J Foot Ankle Res. 2017 Dec 15;10:57. doi: 10.1186/s13047-017-0240-3 (PMC5732456; doi:10.1186/s13047-017-0240-3)
Supplement: Supplementary file 3 — Additional Notes on Results. (DOCX 21 kb) [file 13047_2017_240_MOESM3_ESM.docx]

**Journal of foot and ankle research**

**Additional file 3**

**Identifying the primary outcome for a randomised trial in rheumatoid arthritis: the role of a discrete choice experiment.**

**Eugena Stamuli^1^, David Torgerson^1^, Matthew Northgraves^1^, Sarah Ronaldson^1^, Lindsey Cherry^2^**

1. York Trials Unit, Department of Health Sciences, University of York, York YO10 5DD, UK

2. Solent NHS Trust & University of Southampton, Faculty of Health Sciences, B45, Southampton, SO17 1BJ, UK

**Corresponding author**

Eugena Stamuli

[eugena.stamuli@york.ac.uk](mailto:eugena.stamuli@york.ac.uk)

Tel: +44(0)1904 321871

**Additional Notes on Results**

## The models

### Conditional logit

Two analyses were conducted. In the first one, a constant variable depicting whether the profile was the left or the right hand one in the choice set was included in the model specification. The constant, in this case, has no natural interpretation; it could only reveal whether respondents had a preference towards the left or right hand scenario (some sort of lexicographic preference) and it is expected to be zero (i.e. the choices of the respondents are based on the actual attributes included in each choice set). Indeed, the analysis revealed that the constant is not significant (-0.023, CI: -0.172, 0.126). Hence, only the results of the analysis without including the constant are presented in Table 5.

### Mixed logit

The results of the mixed logit model are presented in Table 4 of the main paper. For the attributes where the standard deviations around the coefficients are statistically significant, this denotes that there is evidence of unobserved preference heterogeneity in the data [[1](#_ENREF_1)] i.e. some respondents show higher interest towards certain attributes compared to other respondents. It was not possible to fit a model with correlated attributes as the models did not converge.

### Generalized multinomial logit

None of the S-MNL models were significant, based on the Wald test. The prob>chi2 values for S-MNL with fixed ASC, S-MNL with random ASC, and S-MNL without ASC were 0.126, 0.34, 0.44 respectively (14, 14 and 13 degrees of freedom respectively). Hence, we cannot reject the null hypothesis that all of the regression coefficients across both models (null and estimated model) are simultaneously equal to zero. This outcome could indicate that the scale heterogeneity is not a major concern for this dataset.

The GMNL model with uncorrelated coefficients did not converge. Computational issues could be the reason for this. For example, by increasing the number of draws from 500 to 1000, there was a 30% decrease in the prob>chi2 (0.2451 from 0.349). However, it is highly possible that this specification was not appropriate for our data i.e. the concurrence of preference and scale heterogeneity does not exist in this dataset. Indeed, the lack of scale heterogeneity was established while attempting to run the S-MNL models.

References

1. Gu Y, Hole A, Knox S: **Fitting the generalized multinomial logit model in Stata.** *Stata J* 2013, **13:**382-397.
